# COVID-19 Vaccination: The Mainspring of Challenges and the Seed of Remonstrance

**DOI:** 10.3390/vaccines9121474

**Published:** 2021-12-13

**Authors:** Hoda Najjar, Hadeel T. Al-Jighefee, Abeer Qush, Muna Nizar Ahmed, Sara Awwad, Layla Kamareddine

**Affiliations:** 1 Department of Biomedical Science, College of Health Sciences, QU Health, Qatar University, Doha P.O. Box 2713, Qatar; hn1517144@student.qu.edu.qa (H.N.); ha1510267@student.qu.edu.qa (H.T.A.-J.); 200050311@student.qu.edu.qa (A.Q.); ma1507488@student.qu.edu.qa (M.N.A.); sa1900788@student.qu.edu.qa (S.A.); 2 Biomedical Research Center, Qatar University, Doha P.O. Box 2713, Qatar; 3 Biomedical and Pharmaceutical Research Unit, QU Health, Qatar University, Doha P.O. Box 2713, Qatar

**Keywords:** SARS-CoV-2, COVID-19, vaccination, challenges, production, distribution, remonstrance, safety, public perception

## Abstract

As of March 2020, the time when the coronavirus disease 2019 (COVID-19) caused by the severe acute respiratory syndrome coronavirus 2 (SARS-CoV-2) became a pandemic, our existence has been threatened and the lives of millions have been claimed. With this ongoing global issue, vaccines are considered of paramount importance in curtailing the outbreak and probably a prime gamble to bring us back to ‘ordinary life’. To date, more than 200 vaccine candidates have been produced, many of which were approved by the Food and Drug Administration (FDA) for emergency use, with the research and discovery phase of their production process passed over. Capering such a chief practice in COVID-19 vaccine development, and manufacturing vaccines at an unprecedented speed brought many challenges into play and raised COVID-19 vaccine remonstrance. In this review, we highlight relevant challenges to global COVID-19 vaccine development, dissemination, and deployment, particularly at the level of large-scale production and distribution. We also delineate public perception on COVID-19 vaccination and outline the main facets affecting people’s willingness to get vaccinated.

## 1. Introduction

In late 2019, the first coronavirus disease 2019 (COVID-19) cases, caused by the novel severe acute respiratory syndrome coronavirus 2 (SARS-CoV-2), were reported in Wuhan, the capital of Hubei province, in Central China. Since then, and until 29 June 2021, this global pandemic has emanated more than 181 million confirmed COVID-19 cases and claimed around 4 million lives [1]. To halt the ongoing disease spread, fledgling measures including maintenance of social distancing, hygiene practices, and the use of repurposed drugs have been held [2]. Despite these taken measures; however, the COVID-19 imposts still grew [3,4], with particular menace to the elderly [5] and patients with chronic illness, mainly those suffering from respiratory and cardiac disorders, obesity, diabetes, and chronic kidney disease [6]. In addition to the endowment of the patient’s underlying health condition to the COVID-19 toll, the viral load was among the chief factors ruling the clinical course of the disease [7]. Currently, the stage has been set for diverse therapeutic approaches to take part in governing the infection as one disease control strategy. In this context as well, and as one avant-garde attempt to control the pandemic and prevent future outbreaks, the World Health Assembly called attention to the role of immunization in averting, restraining, and winding up COVID-19 [8]. Pinning hopes on vaccines to prevail over the disease roll out stemmed from the success of vaccine weapons developed against the spread of previous infectious diseases [3]. So far, more than 200 COVID-19 vaccine candidates are available, with 105 candidates currently in the clinical phase and 184 in the pre-clinical phase of development [9]. Of those, 18 vaccines are to date approved by at least one country for emergency use [3,9,10,11]. Generally, producing newly licensed vaccine entails a multi-linear series of steps stretching over several years. Yet, the sudden outbreak of COVID-19 necessitated swift action and unmatched pharmaceutical and non-pharmaceutical endeavors to develop a safe and effective vaccine in a terse time frame [12], with the research and discovery phase of the vaccine development process being skipped [13,14,15]. Producing vaccines at such an unprecedented time and leaping over a fundamental stage in the development process could impose huge challenges at the level of development, dissemination, and deployment, three practices designated by Forman et al. as the “three D’s” [16]. This practice of vaccine production is also expected to bring in vaccination hesitancy and affect the public’s willingness to get vaccinated. In this review, we delineate the challenges acquainting the “three D’s” of effectuating omnipresent immunity against COVID-19 via vaccination [16] and set forth the paradigm in COVID-19 vaccine hesitancy as brought by several established vaccine hesitancy and acceptance theoretical models [10,17,18,19,20,21,22,23].

## 2. Challenges Obstructing Global COVID-19 Vaccine Rollout

### 2.1. Challenges in Vaccine Development

**Figure 1:**
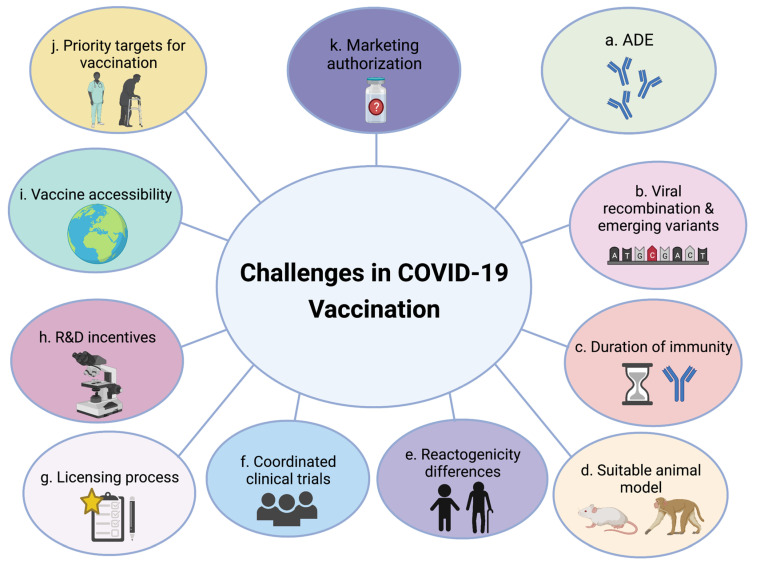
Challenges in COVID-19 Vaccination. (**a**) Virus-specific antibody-dependent enhancement (ADE) disease. (**b**) Impact of viral genetic recombination, high mutation rates, and emerging viral variants on vaccine effectiveness. (**c**) Duration of vaccine-acquired immunity. (**d**) Utilization/availability of suitable animal models to conduct vaccine safety and efficacy studies. (**e**) Differences in reactogenicity between age groups. (**f**) Running coordinated and valid vaccine clinical trials. (**g**) Facilitated and synchronized regulatory licensing processes. (**h**) Continuous research and development (R&D) incentives. (**i**) Public vaccine accessibility. (**j**) Deciding on priority target populations for vaccination. (**k**) Obtaining vaccine marketing authorization. The figure was created with BioRender.com.

A significant consideration in vaccine development is the occurrence of antibody-dependent enhancement (ADE) disease upon vaccination (Figure 1a). Although this phenomenon has not been well outlined yet, virus-specific antibody production can, under certain conditions, enhance the rate of viral infection or trigger immunopathology [24,25,26,27]. Cases of pulmonary immunopathology associated with Th2 response were previously reported in SARS-CoV and the Middle East respiratory syndrome (MERS-CoV) vaccine studies conducted in mice models [28,29]. Although clinical trials of COVID-19 vaccine candidates have not presented evidence of ADE thus far, the issue remains obscure herein and necessitates a well-regulated T-cell response as an essential element for efficient SARS-CoV-2 vaccine development [27]. Genetic recombination and high mutation rates culminating in variants possessing enhanced virulence and the capability to escape immune recognition is another main challenge faced during vaccine development against viruses [30,31,32]. With the ongoing COVID-19 pandemic, mutations in the SARS-CoV-2 spike (S) protein have occurred [33], reducing virus neutralization potency [34]. Besides this, the vaccine’s ability to show effectiveness against more virulent/newly emerging viral strains is another raised concern (Figure 1b) [34]. Also, much remains unknown about the duration of vaccine-acquired immunity, particularly the short and long-term safety and efficacy of most COVID-19 vaccines (Figure 1c). It was initially thought that undetectable levels of infection-induced antibodies against SARS-CoV-2 will be achieved within a few months following the disease [35]. However, recent serological studies have reported a constant level of neutralizing antibodies (nAbs) against the receptor-binding domain (RBD) of the S protein and S2 subunit for 5–7 months following infection as compared to a rapid waning of nAbs titers against the nucleocapsid (N) protein [35]. Except for men, older frail individuals, and individuals with comorbidities, vaccine effectiveness against severe disease conditions was shown to persist for almost 9 months, but at different rates varying with the vaccine type [36]. Conversely, previous similar studies on SARS-CoV revealed detectable nAbs titers for up to 12–17 years following infection [37], with long-lasting reactive memory T-cells against the N protein for 17 years after infection [38]. Amongst those studies, many viral reactivation or reinfection cases have been reported in various countries [39,40,41,42,43,44], with the second infection being more severe than the first in several occurrences, particularly those in the United States and Ecuador [32]. Fortunately, and as evidenced by released World Health Organization (WHO) reports and novel study findings, at least some of the developed COVID-19 vaccines are still expected to provide a degree of protection against the new emerging variants, particularly those capable of eliciting a broad immune response [45,46,47]. Yet, the produced nAb titers are relatively lower than those produced against the ancestral strain [46,47,48]. Moreover, and until now, not all approved vaccines have been tested against all these variants, making a conclusive evaluation of the effectiveness of the current vaccine candidates difficult at this stage. As conjectured, in scenarios of reduced vaccine effectiveness against the new variants, approaches including an additional booster dose or a modified vaccine with variant-specific proteins could provide better potency. In July 2021, Pfizer announced a plan for a booster dose of its COVID-19 vaccine to enhance protection against the new variants including the delta variant, which showed reduced sensitivity to antibody neutralization [49,50]. The proposed plan was; however, curbed due to the lack of enough evidence supporting the population’s need for a third dose, especially that half of the US population at that time was not vaccinated yet [50,51]. The use of a third dose of Pfizer-BioNtech vaccine was, instead, endorsed for those who are 65 years and older or those at high risk of severe disease [52]. Likewise, Moderna announced a third booster dose and booster vaccine candidates as a counter variants plan [48], particularly after reporting a six-fold reduction in the nAb titers against the South African variant (B.1.351) [34,53]. Taken to the implementation stage, a booster shot of Moderna vaccine for adults over 65 years of age and for people at high-risk such as healthcare workers has been set. Unlike the adopted practice for Pfizer; however, Moderna’s booster jab will be only half a dose (50 micrograms), with a timeframe of at least six months after the second dose. The rationale of this dose reduction derives from the intention of reducing the risk of potential side effects associated with getting vaccinated [54,55]. Should the COVID-19 pandemic remain uncontrolled, booster shots for all vaccinated individuals might be put back on the table.

Interestingly, recent data on many of the currently approved COVID-19 vaccine candidates for emergency use show that most of these vaccines not only protect against COVID-19 but also against other severe diseases [56]. Taking this into account, and in quest of establishing sterilizing immunity, significant efforts are now being employed to ensure that the majority of the population gets vaccinated [57]. Studies on non-human primates, showed that most COVID-19 vaccine candidates provide complete or partial protection following viral challenge in the upper and lower respiratory tract [26,58,59]. Similarly, other studies on newly developed vaccines revealed a protective outcome in the upper respiratory tract when vaccines are administered at higher doses [27]. Achieving sterilizing immunity in the upper respiratory tract may be essential to prevent the spread of the virus [27,60]. As such, intranasal vaccine platforms directed towards inducing a strong mucosal immune response in the upper respiratory tract are now being investigated [27,59]. In May 2021, a recent study reported that a single-dose of an intranasal COVID-19 vaccine candidate(AdCOVID) could provide sterilizing immunity in the lungs of vaccinated animals [61]. This was also evident by the generation of potent serum- nAb responses, T cell responses, and robust induction of mucosal immunity in vaccinated mice. Unfortunately however, recent updates from AdCOVID phase 1 clinical trial showed no adequate stimulated immune response in healthy volunteers, driving the termination of this intranasal vaccine development process [62]. Despite these discouraging updates, intranasal vaccines still have tantalizing promises due to their ability to elicit mucosal immunity at the linings of the nose and lungs, which would protect not only from infection but also from transmission. Other attributes advocating the development of such novel vaccine platforms with fostered vaccine acceptance among populations is them being needle-free, thermostable, and delivered in a single dose [61]. In addition to its ability to induce potent and protective immune responses against SARS-CoV-2 infection in a safe manner, an important indicator of vaccine effectiveness is the generation of memory cells that can recognize the pathogen and quickly neutralize it upon subsequent exposure. In most vaccine trials, the binding ability and neutralizing antibody titers are used as benchmarks for efficacy assessment, although they provide limited insight on the protective immunity against COVID-19 [63]. It is worth mentioning here that our understanding of the protective ability of nAbs against SARS-CoV-2 infection and the ability of the newly developed COVID-19 vaccines to limit viral spread is still primitive.

Typically, several immune mechanisms, including both cellular and humoral responses, are triggered within an individual following infection or vaccination [64]. In a SARS-CoV-2 infection setting, reports have shown that specific and durable T-cell immunity is elicited against multiple protein targets (or epitopes), with a broad diversity of alpha (B.1.1.7), beta (B.1.351), and gamma (P.1) variants recognition [38,63,65]. Yet, a recent report from Public Health England revealed that34.9% of people hospitalized with delta cases between July and August have been administered two doses of COVID-19 vaccine [66]. This raises concerns regarding the ability of COVID-19 vaccines to confer sterilizing immunity or at least provide sufficient immunity against the delta variant or other possibly upcoming variants. Furthermore, and due to the vast variability among reported data, a better understanding of the immune responses generated after SARS-CoV-2 infection and vaccination, emphasizing on the status of the adaptive immune response and how could this be harnessed to develop COVID-19 vaccines capable of conferring sterilizing immunity, is needed.

A suitable animal model for understanding the mechanism of viral infection in humans and testing the safety and efficacy of the SARS-CoV-2 vaccine is also a major challenge faced in vaccine development (Figure 1d) [27]. In current COVID-19 studies and vaccine clinical trials, many of the used animal models do not precisely mimic the human immune response to the virus, nor do they possess the required receptor found on human cells for virus entry [67]. As such, the mode of action, vaccine effectiveness, and antibody production should be evaluated in humans for accurate gauging of the risks and benefits of the vaccine [24,27]; an entail fulfilled by obtaining the needed authorization and by the willingness of human participants to take part in ongoing studies and clinical trials. With such a dearth in carried out in vivo studies, differences in reactogenicity between the age groups following vaccine administration and immunization also emerges as a chief concern (Figure 1e) [27]. This is primarily owed by the fact that most vaccine clinical trials have enrolled healthy adults between the ages of 18–55 years old, while very few trials (in their later stages) were conducted on participants in older age groups (over 55 years of age) [27]. Moreover, minors under the age of 18 years were also not initially enrolled in clinical trials [68,69].

To boot, running coordinated and valid clinical trials at an unparalleled pace is another challenge contemplated in the COVID-19 vaccine development process (Figure 1f), with a number of facets to be pondered in such a ‘race against time’ operation. In addition to the importance of methodology and communication in conducting efficient clinical trials, sample management appears among the foundations. Taken the fact that the COVID-19’s burden predominantly afflicts the older and minority ethnic populations, as well as those suffering from co-morbidities [70,71,72,73] it is crucial for vaccine clinical trials to enroll all categories of the general population who will be receiving the vaccines when successfully developed, authorized, and approved in the market [27]. Backlash, particularly that relevant to limited age, racial, and ethnic diversity among trial participants, has acquainted several vaccine platforms clinical trials, holding a potential risk of hindered trust in the generated vaccines [72]. Facilitated and synchronized regulatory licensing processes for hastened vaccine approval are also among the other relevant concerns arising during vaccine development (Figure 1g) [16,74].

A remarkable degree of collaborative efforts has made the accelerated authorization of COVID-19 vaccines possible [16], with approximately 15% of the world’s population until now being fully vaccinated [75]. The need of an additional tremendous number of doses to vaccinate the remaining billions [16]; however, along with the ambiguity revolving around the ability of the current first-generation vaccines to provide immunity against the newly emerging variants [76], the duration of vaccine-acquired immunity, and the possible need of booster doses [16], necessitates constant research and development (R&D) incentives for manufacturers. Continuous R&D ensures regular investigation of viable vaccine platforms [77] and therefore helps in achieving COVID-19 long-term solutions (Figure 1h). Research systems should be also established to identify arising viral mutations and continuously share gene sequencing data to modify existing vaccines as needed and develop alternatives if necessary [16].

### 2.2. Challenges in Vaccine Dissemination and Deployment

Mass production, innovative solutions to increase manufacturing compass, and proper control of the supply chain capacity and distribution, are among the major demands required for adequate availability and, therefore, equitable accessibility of vaccine doses to everyone across the globe [15].

Woefully though, the process of vaccine distribution is not always paved and sometimes shapes as a footrace for ample and fair access to the vaccine. The financial ability of governments to afford developing/buying vaccines appears as a top strand for vaccine public accessibility (Figure 1i). Manufacturing enough quantities and maintaining supply chain capacity are among the primary challenges faced herein, particularly for low middle-income countries (LMICs) [16,78,79]. Furthermore, cold chain supply and other complex supply chain systems appear as an additional challenge for countries with poor infrastructure. Approximately 20% of the poorest countries are not equipped with an appropriate cold chain capacity [16,80,81,82], whereas those that do have such resources remain unable to keep vaccines cool due to certain equipment malfunctions [16]. Along those lines, the economic disparity between countries could have high-income countries monopolize global vaccine supply, a scenario encountered during the 2009 influenza A (H1N1) pandemic where developed countries placed large vaccines orders in advance, leaving the impoverished countries undersupplied [83,84,85,86]. A “globally fair allocation system” financed by the public sector and composed of global purchasing agent(s) and advanced purchase commitments could, for example, help in overcoming the cost barrier problem. Such a system translates into providing free vaccines for prioritized populations at the point of care worldwide, followed by a fair and objective national allocations process [85] that takes into account racial, social, and ethnic factors when determining vaccine distribution strategies [87,88,89].

Should such strategies be successfully executed, integral facets could still reside in the implementation phase of vaccination programs, peculiarly those pertinent to identifying the main goal of vaccination in either preventing mortality and easing the disease burden or curbing the pathogenic spread. Deciding on the priority target of being vaccinated is also challenging and depends on several factors including the population’s demographics, the prevalence of the disease, the government’s budget for obtaining or developing the vaccine, and the available vaccine supply (Figure 1j) [90,91,92]. In cases where the primary goal of vaccination is to reduce death rates and mitigate the disease burden imposed on healthcare systems, the priority is given to high-risk groups such as the elderly, frontline healthcare workers, and individuals suffering from chronic diseases such as hypertension, diabetes, cardiovascular diseases, respiratory diseases, kidney diseases and obesity [15,90]. Should the vaccines be available in sufficient quantities; however, with the target being to control disease transmission, vaccination strategies should also involve younger age groups, including those that could be asymptomatic. To consider such a scenario though, a vaccine should not only prove effectiveness in protecting individuals from severe symptomatic disease, but also demonstrate the ability to prevent the occurrence and transmission of the infection [15].

Another challenge likely faced during the vaccine implementation phase is obtaining marketing authorization post-assessment of the submitted vaccine dossier, particularly that relevant to vaccine effectiveness and side effects [15,93,94]. Owing to our constantly changing fundamental understanding of the SARS-CoV-2 virus characteristics, evaluating the efficacy and safety of COVID-19 vaccines for marketing authorization might not be easy though and is therefore gauged as a complex and challenging process [15,93] (Figure 1k). Should this process successfully pass the marketing authorization stage, concerns regarding suitable vaccine storage temperatures, the need of sufficient resources, and the availability of dedicated and adequately trained personnel to participate in the vaccination campaign eventually pitch in as additional challenges [16].

## 3. Monads of COVID-19 Vaccination Hesitancy

**Figure 2:**
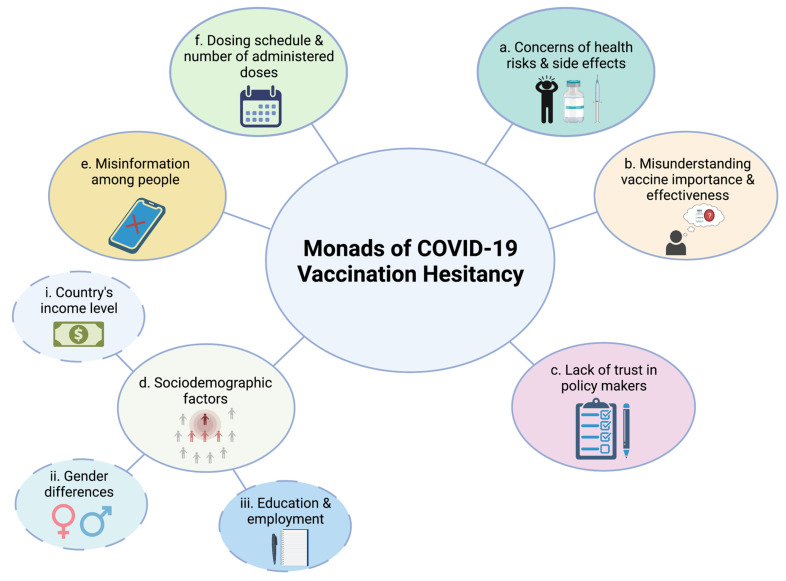
The Monads of COVID-19 vaccination hesitancy. (**a**) Apprehension due to presumed health risks and inaccurate knowledge regarding the vaccine’s side effects. (**b**) Improper understanding of the importance and effectiveness of the vaccine. (**c**) Lack of trust in policy makers. (**d**) Sociodemographic factors attributing to variations in vaccine acceptance rates including (**i**) country’s income level, (**ii**) gender differences, and (**iii**) education level and employment status. (**e**) Inaccurate and incomplete information about vaccines provided to the public. (**f**) Dosing schedule and the number of administered doses. The figure was created with BioRender.com.

Vaccine refusal is not a new phenomenon exclusive to the COVID-19 pandemic. Indeed, the trepidation of being vaccinated was already a fattening concern prior to the COVID-19 pandemic [22], with several challenges arising in implementing community immunization, particularly those relating to the societies’ and individuals’ recognition of vaccines as necessary, safe, and effective [95,96]. During the 2009 H1N1 pandemic, for example, vaccine hesitancy was inferred from the low vaccination rates ranging between 0.4 to 59% in 22 countries [97,98,99]. Vaccination hesitancy, which is usually considered a composite and dynamic social circumstance [18], could be simply presented in a framework encompassing several building blocks [10,17,18,21,22,23]. Concerns relating to alleged health risks and inaccurate knowledge on the vaccine’s side effects (Figure 2a), improper understanding of the importance and effectiveness of the vaccine (Figure 2b), and lack of trust in policy makers (Figure 2c), are among the major building blocks of vaccination hesitancy as they can significantly hinder efforts directed towards establishing herd immunity [22,100]. Fostering COVID-19 vaccine uptake is primary subject to people’s readiness to get vaccinated. Several sociodemographic factors (Figure 2d), including gender, race, ethnicity, age, educational level, employment status, and religious beliefs could also contribute to vaccine hesitation. Vaccine acceptance rates could, for instance, vary with the country’s income level (Figure 2d-i). As an example, a study by Solís Arce et al. reported a higher willingness to take the COVID-19 vaccine in people living in low- and middle-income countries (LMICs) compared to those living in the United States and Russia [101]. The life experience of people in LMICs could conceivably explain vaccine acceptability in these countries with thousands of deaths yearly recorded in LMICs from vaccine-preventable infectious diseases [10]. Into the bargain, studies conducted in several countries show that males are more receiving to COVID-19 vaccine than females (Figure 2d-ii) [102,103]. The educational level and employment status of individuals may also distort their understanding of the safety, efficacy, and necessity of vaccination. People with a higher degree of education and a full-time job are thought to better advocate vaccination (Figure 2d-iii) [103]. A considerable level of vaccine rejection could be also attributed to insufficient, incomplete, or inaccurate information provided to the public by various means including social media platforms (Figure 2e) [104]. Denial to vaccinate children, for instance, could be due to lack of available data on the younger population’s response to the vaccine [105,106,107,108], along with the circulating misinformation and fabricated reports on vaccine safety in children [104]. Another driver for vaccine hesitancy among populations is the anti-vaccination movements. Many of these movements spread conspiracy theories and misinformation about COVID-19 vaccine safety, a practice that caused worrisome-based reduction in COVID-19 vaccination rates worldwide [109], particularly during the early phases of the pandemic when vaccines were first introduced. It is worth noting here that such anti-vaccination movements have been growing over the past decades and have caused similar declines in vaccination rates and the potential resurgence of other diseases such as measles. A major factor contributing to the influence of these activists on people is their increased access to social media platforms and some official government websites [110,111]. Despite the aforementioned impact of anti-vaxxers, mainly during the early phases of the pandemic, their current hype on the COVID-19 vaccines rollout seems to be of no significant effect, given that approximately 52.8% of the global population have so far received at least a dose of the COVID-19 vaccine [112]. This could be attributed to an increase in the population awareness, especially in high-income countries, about the benefits and importance of vaccination. Also, those who tested positive for SARS-CoV-2, or had an infected family member are probably now more likely to accept vaccination than before [113].

The number of administered vaccine doses and the dosing schedule, particularly in LMICs, are also among the plausible factors that could affect the people’s willingness to get vaccinated (Figure 2f). The availability of single-dose vaccines, for example, could be advantageous in settings of high vaccination demands yet relatively low-capacity healthcare systems [101]. The need of several doses might also have people question the vaccine’s efficacy and reliability to induce protection against SARS-CoV-2 infection. Indeed, surveys have shown that hesitant respondents were mainly concerned about vaccine efficacy along with its potential side effects [101]. A directed practice towards mixing vaccine types [114] and providing booster doses [115] may also play a role in vaccine hesitancy.

## 4. Conclusions

With the under-way SARS-CoV-2 pandemic, a potent and safe vaccine could be the key in fighting the COVID-19 battle. Though imperative, considerable logistics and social challenges have been faced since the COVID-19 vaccine rollout. Despite the pronounced triumph in delivering legion vaccine candidates into the market, a paved expedition from vaccine revelation to world-wide herd immunity is still far beyond reach. Strategized solutions and framed out action plans are required to overcome the encountered COVID-19 vaccination challenges, not only to sail safely away from the current pandemic but also to avert succeeding future sways.

## Data Availability

Publicly available resources for information used in the review are in-text cited as relevant.
